# Two chemotherapeutic agents expand stem-like CD62L^+^CD8^+^ T cells in antitumor immune responses

**DOI:** 10.3389/fimmu.2025.1533857

**Published:** 2025-04-01

**Authors:** Xiaokang Ruan, Linwei Wu, Zijian Tang, Yao Li, Jin Wang, Haolin Jiang, Li Zhang, Shengjia Wang, Zhaoqiang Chen, Chenlei Yuan, Yujian Xia, Yan Pan, Jianling Gao, Xin Zhao

**Affiliations:** ^1^ Department of General Surgery, The First Affiliated Hospital of Soochow University, Suzhou, China; ^2^ Department of General Surgery, People's Hospital of Dongxihu District, Wuhan, China; ^3^ The Affiliated Infectious Diseases Hospital, Suzhou Medical College of Soochow University, Suzhou, China; ^4^ Department of Gastroenterology, The First Affiliated Hospital of Soochow University, Suzhou, China; ^5^ Department of General Surgery, The Fourth Affiliated Hospital of Soochow University, Suzhou, China; ^6^ Jiangsu Institute of Clinical Immunology, The First Affiliated Hospital of Soochow University, Suzhou, China; ^7^ Jiangsu Key Laboratory of Clinical Immunology, Soochow University, Suzhou, China; ^8^ Jiangsu Key Laboratory of Gastrointestinal Tumor Immunology, The First Affiliated Hospital of Soochow University, Suzhou, China; ^9^ Department of Critical Care Medicine, The Fourth Affiliated Hospital of Soochow University, Suzhou, China

**Keywords:** chemotherapeutic agents, CD62L^+^ CD8^+^ Tpex cells, CD8^+^ TTSM cells, Eomes, antitumor immune responses

## Abstract

**Introduction:**

Recent findings reveal that the precursors of exhausted CD8^+^ T (CD8^+^ Tpex) cells possess stem-like signatures in tumor immunity, which originate from tumor draining lymph node (TdLN)-derived tumor-specific memory (CD8^+^ T_TSM_) cells. Both of these T subsets can be collectively referred to as stem-like CD8^+^ T cells, which demonstrate robust self-renewal ability and can proliferate and differentiate into transitory effector-like exhausted T cells (Tex^int^). There are reports that chemotherapeutic drugs can promote the antitumor immune responses of patients by increasing the number of CD8^+^ T cells; however, whether chemotherapeutic drugs increase these two stem-like CD8^+^ T cells remain further exploration.

**Methods:**

Tpex cell-associated subpopulations in human colorectal tumors were analyzed by using single-cell sequencing data. CT26 and B16 tumor models of wild type and Eomes conditional knockout mice were constructed, and the changes of T_TSM_, Tpex and Tex subsets in mice were dissected by flow cytometry after treatment with decitabine (DAC), doxorubicin (DOX) and 5-Fluorouracil (5-FU).

**Results:**

In this study, we demonstrated that DAC and 5-FU expanded CD8^+^ T_TSM_ cells in TdLNs. At the same time, we validated that DAC and 5-FU substantially promoted the expansion of CD62L^+^CD8^+^ Tpex cells and subsequently increased effector function of CX3CR1^+^ CD8^+^ Tex^int^ cells. In addition, the conditional knockout of transcription factor Eomes in CD8^+^ T cells partially eliminated DAC-amplified CD62L^+^ CD8^+^ Tpex cells, but had no effect on such CD8^+^ T subset expanded by 5-FU.

**Conclusion:**

The present study demonstrated that both DAC and 5-FU promoted the differentiation of stem-like CD8^+^ T_TSM_ cells in TdLNs and significantly enhanced the differentiation and expansion of stem-like CD62L^+^ CD8^+^ Tpex and CX3CR1^+^ Tex^int^ cells in tumor microenvironment. The knockout of Eomes partially influenced the role of DAC in promoting the differentiation and expansion of stem-like CD8^+^ T cells.

## Introduction

Persistent antigen stimulation leads to CD8^+^ exhausted T cells (Tex), with upregulation of programmed death-1 (PD-1) and other inhibitory receptors, perturbed proliferation and cytokine secretion, impaired immune memory, and altered metabolism (1). CD8^+^ Tex cells are not only distinctively characterized in terms of function, metabolism, transcription, and epigenetics but also form a heterogeneous cell population. As a result, a CD8^+^ T cell subset that retains stemness and memory potential has been identified as a crucial element in response to immune checkpoint blockade (ICB) and other immunotherapies (2–4). We, and others, have demonstrated that intratumoral CD8^+^ stem-like T cells are tumor-antigen specific, exhibit multifunctional effector capacities, and generate a proliferative burst that fuels the effector response. Despite evidence suggesting that transcription factor (TF) TCF-1-expressing CD8^+^ Tpex cells and CD8^+^ T_TSM_ cells are primary responders to PD-1/PD-L1 ICB, further investigation is required to confirm this finding (5–7). CD8^+^ T_TSM_ cells in tumor-draining lymph nodes (TdLNs) can differentiate into CD8^+^ Tpex cells [tumor-draining lymph node (TdLN)-CD8^+^ Tpex] and gradually migrate to the tumor microenvironment where they become TME-CD8^+^ Tpex cells, further differentiating into transitory effector-like exhausted T cells (Tex^int^) cells that sustain antitumor effects (6).

The advent of ICB therapy has marked a significant advance in cancer treatment but fails to elicit durable clinical benefit in many patients. Conventional chemotherapeutic agents can selectively destroy malignant cells due to their accelerated replication rate and thus may serve as clinically useful agents. Nevertheless, the clinical efficacy of diverse chemotherapeutic agents is contingent upon the stimulation of antitumor immunity, either by triggering the discharge of immunostimulatory molecules from apoptotic malignant cells or by eliciting off-target effects on immune cell populations (8). DNA methylation plays a pivotal role in epigenetic gene regulation, facilitating the terminal differentiation of Tex cells (9). Low-dose decitabine (DAC), a DNA hypomethylating agent, can augment the activation and cytolytic activity of CD8^+^ T cells (10). Also, the primary administration of 5-FU can alter the distribution of MDSCs, DCs, and T lymphocytes within the tumor microenvironment; Type I conventional dendritic cells (cDC1) form a CD8^+^ T cell niche that sustains and guides Tpex cell differentiation (11). A comprehensive understanding of the potential mechanisms of the action of chemical drugs on stem-like CD8^+^ T cells and ways to induce high reactivity and long-term efficacy of stem-like CD8^+^ T cells is crucial.

This study was conducted to investigate the role of chemotherapies in priming tumor-antigen-specific CD8^+^ T_TSM_ cells in TdLNs and in shaping the intratumoral CD8^+^ Tpex cell response across various tumor models. We used flow cytometry and identified the significantly expanded CD62L^+^CD8^+^ Tpex cell subset after the treatment with chemotherapeutic agents (DAC and 5-FU). Also, we preliminarily explored the potential molecular mechanisms of stem-like CD62L^+^ CD8^+^ T cells mediated by chemotherapeutic agents.

## Results

### Stem-like CD62L^+^CD8^+^ Tpex subset was found in colorectal cancer

**Figure 1:**
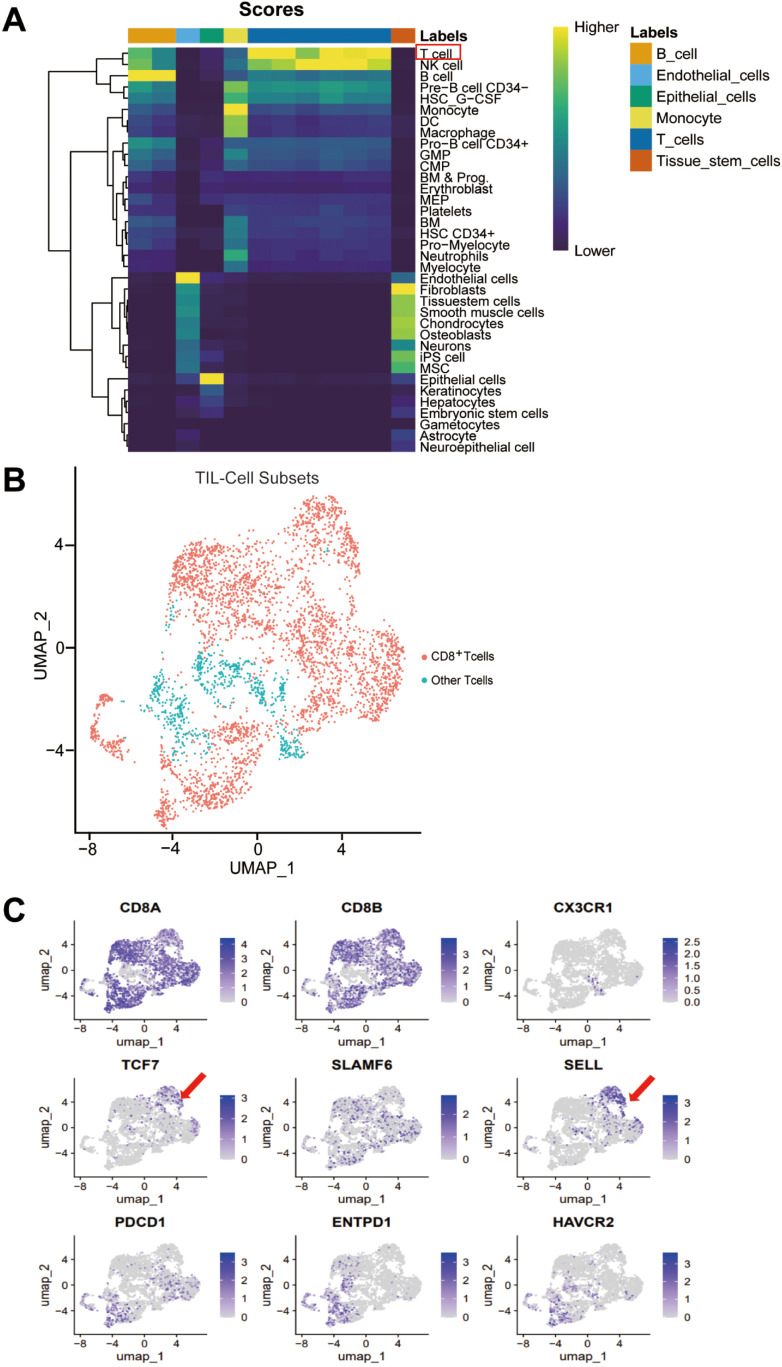
Stem-like CD62L^+^CD8^+^ Tpex subset was found in colorectal cancer. Single-cell sequencing data of human colorectal cancer tumor specimens were obtained from the GEO database (GSE200997) (*n* = 23). **(A)** Heatmap of immune cell clustering in the microenvironment of human colorectal cancer tumors. **(B)** UMAP showing the distribution of CD8^+^ TILs in human colorectal cancer tumors. **(C)** UMAP of differential gene enrichment in various subpopulations of CD8^+^ TILs in human colorectal cancer.

This study acquired the single-cell sequencing data of 23 human colorectal cancer samples from the GEO database (GSE200997). First, the immune cells in human colorectal cancer were analyzed using the clustering heatmap to find out the tumor-infiltrating lymphocytes (TILs) marked by red boxes (**Figure 1A**), followed by UMAP downscaling to analyze CD8^+^ TILs distributed in the red region in the UMAP map of T lymphocytes (**Figure 1B**). Further analysis of differential gene enrichment on the UMAP map of CD8^+^ TILs revealed a distinct subpopulation of stem-like CD8^+^ Tpex cells. These cells exhibited high expression of TCF-1 (encoded by *Tcf7*) and CD62L (encoded by *Sell*), along with reduced expression of PD-1 (encoded by *Pdcd1*) (**Figure 1C**). This subpopulation was significantly different from the transition-state CD8^+^ Tex^int^ cells, characterized by high *Cx3cr1* expression, and terminally exhausted CD8^+^ Tex^term^ cells, which highly expressed *Entpd1* and *Havcr2* in colorectal cancer; it was in line with the results of related studies (12).

The aforementioned findings indicate that a population of stem-like CD62L^+^ Tpex cells exists in human colorectal cancer tissues. Therefore, we need to precisely analyze and validate the molecular phenotype of stem-like CD62L^+^ Tpex cells, and their differentiation and mechanisms through *in vivo* animal experiments. We also should explore the new pathways of chemotherapeutic agents to promote the differentiation of stem-like CD62L^+^ Tpex cells.

Clinical studies have confirmed that some of chemotherapeutic agents can mediate the antitumor immune response in patients by increasing the proportion and number of CD8^+^ TIL cells in the tumor microenvironment (13, 8, 14). Based on the findings shown in **Figure 1**, it is hypothesized that the antitumor effects of chemotherapeutic drugs may be closely related to CD8^+^ stem-like T cell differentiation.

### Chemotherapeutic agents restrained the progression of colorectal cancer and melanoma and increased the number of CD8^+^ TILs and their effector function

**Figure 2:**
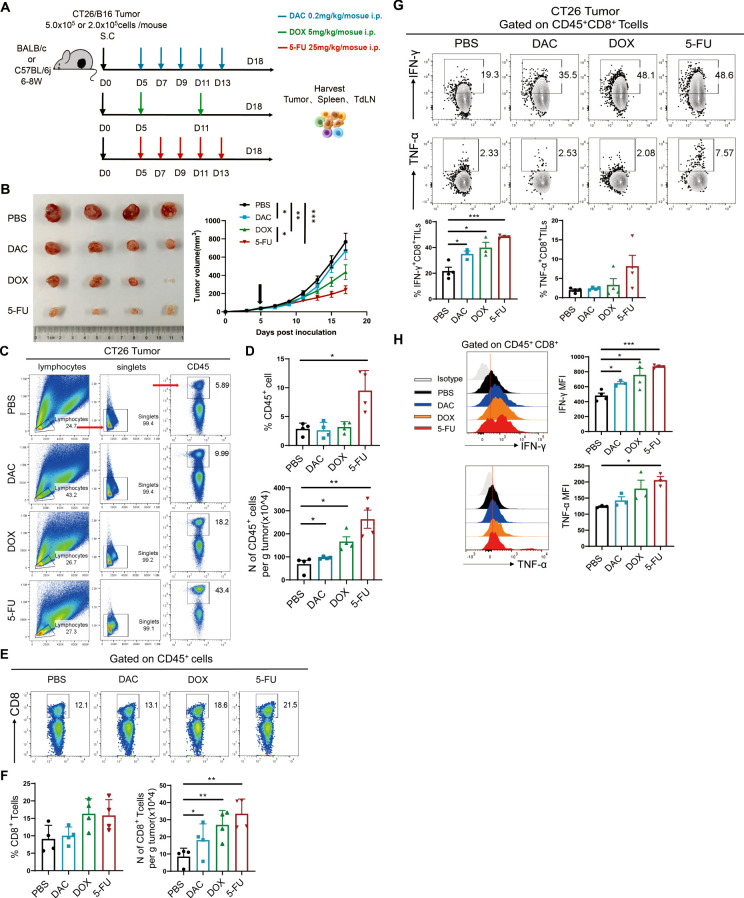
Chemotherapeutic agents restrained the progression of colorectal cancer and melanoma and increased the number of CD8+ TILs and their effector function. **(A)** Experimental procedure of chemotherapy for CT26 colorectal cancer or B16 melanoma. **(B)** Diagram and tumor growth graph of CT26 tumors. **(C, D)** Representational plot, statistical plot, and absolute number of CD45+ immune cells in tumor tissues in CT26 colorectal cancer. **(E, F)** Flow and statistical plots of the absolute number of CD8+ T cells in tumors. **(G)** Flow and statistical plots of CD8+ TILs cellular effectors IFN-γ and TNF-α in tumors. **(H)** Statistical peak plots of MFI values of CD8+ TIL effector proteins IFN-γ and TNF-α in tumors. The data are shown as mean ± SEM. *P < 0.05, **P < 0.01, and ***P < 0.001, using unpaired *t* test, n ≥ 3.

The present study used two mouse subcutaneous hormonal models of CT26 colorectal cancer and B16 melanoma. The experimental procedure is shown in **Figure 2A**. The dimensions of the tumor were quantified every two days in the tumor growth phase, and the CT26 tumor growth curve was plotted. The results demonstrated that the therapeutic efficacy of 5-FU was superior to that of the phosphate-buffered saline (PBS) control in the CT26 colorectal model (**Figure 2B**). At the same time, the therapeutic effect of DOX and 5-FU was the most significant in the B16 melanoma model (**Supplementary Figure S1A**).

T lymphocytes play an indispensable role in the antitumor immune response. The level of infiltration of the main antitumor immune cells (CD45^+^ immune cells and CD8^+^ TILs) was first detected in the tumor tissues by flow cytometry. The proportion of CD45^+^ immune cells in the chemotherapy group exhibited an upward trend, accompanied by a significant increase in the total number of CD45^+^ immune cells in the CT26 model (**Figures 2C, D**). Meanwhile, 5-FU significantly increased the proportion of CD45^+^ immune cells and the absolute number of tumors per gram in the B16 model (**Supplementary Figures S1B, C**). Further analysis of the proportion of CD8^+^ T cells showed that the absolute number of CD8^+^ T cells per gram of tumor in CT26 tumor models significantly increased in the three chemotherapy groups, with the most significant effect in the 5-FU group (**Figures 2E, F**). Nevertheless, the proportion and absolute number of CD8^+^ TIL cells were markedly elevated in the chemotherapy groups, particularly in the DOX group in the B16 model (**Supplementary Figures S1D, E**). CD8^+^ TIL cells could secrete effector factors such as interferon-γ (IFN-γ) and tumor necrosis factor-α (TNF-α) to kill tumors. The results demonstrated that the expression of IFN-γ increased in the chemotherapy groups (5-FU, DAC and DOX) compared with the PBS control group (**Figure 2G**). The mean fluorescence intensity (MFI) value of IFN-γ significantly increased, with the most notable secretion of the effector in the 5-FU group followed by the DOX group, which was coherent with the tumor growth tendency (**Figure 2H**). The chemotherapeutic agents substantially increased the proportion and number of CD45^+^ immune cells, especially the proportion and number of tumor-infiltrating CD8^+^ TILs, and enhanced the secretion of IFN-γ and TNF-α. These findings indicated that the chemotherapeutic agents could impede tumor progression by facilitating the proliferation or survival of CD8^+^ TILs.

### Chemotherapeutic agents repressed PD-1 expression in CD44^+^CD8^+^ TIL cells in tumor tissues

As the classical immune checkpoint inhibitory molecule, PD-1 is differentially expressed in CD8^+^ T cell subsets (15). Moderate PD-1 expression by CD8^+^ T cells inhibit TCR signaling and CD28 co-stimulatory signaling to prevent the overactivation and rapid exhaustion of CD8^+^ T cells after exposure to antigens (16). PD-1 overexpression can impede the long-term survival and self-renewal of CD8^+^ T cells, which is deleterious to the differentiation and long-term stability of CD8^+^ T cell subpopulations. Flow cytometry was used to detect the expression level of PD-1 in tumor tissues. The results showed that in the CT26 colorectal cancer model, the DAC and 5-FU treatments significantly downregulated the expression of PD-1 and the MFI value in CD44^+^CD8^+^ TILs compared with the PBS control. The 5-FU treatment downregulated the expression of PD-1 most significantly, and the difference was statistically significant in all cases (**Supplementary Figures S2A, B**). We found that CD44^+^CD8^+^ TILs comprised three populations of PD-1^hi^, PD-1^int^, and PD-1^low^ with different PD-1 expression levels. DAC and 5-FU significantly decreased the population of PD-1^hi^ cells and increased the populations of PD-1^int^ and PD-1^low^ cells, whereas no significant difference was observed in the DOX treatment group (**Supplementary Figures S2C, D**). Only DAC treatment significantly decreased PD-1 expression in CD44^+^CD8^+^ TILs in the B16 model (**Supplementary Figures S3A, B**), which decreased the PD-1^hi^ population and increased the PD-1^int^ and PD-1^low^ populations (**Supplementary Figures S3C, D**).

Furthermore, the immunosuppressive molecule PD-1 displayed different degrees of expression in tumor-specific CD44^+^CD8^+^ TIL cells. A moderate or lower level of PD-1 expression in CD44^+^CD8^+^ TIL cells (**Supplementary Figure S2E**) facilitated the long-term maintenance of CD8^+^ TIL cells, thus providing sustained tumor control.

### Chemotherapeutic agents significantly modified the proportion of T_N_, T_CM_, and T_EM_ cells in tumors, draining lymph nodes, and spleen of homozygous mice with melanoma

CD8^+^ TILs undergo gradual apoptosis or depletion during sustained tumor immune response. A small proportion of CD8^+^ T cells are differentiated into antigen-specific memory T cells and survive so that they are rapidly activated and differentiated into effector Teff cells for tumor immune response next time following the same antigenic stimulus (17). Many studies have confirmed that T cells are categorized into naive T cells (T_N_), central memory T cells (T_CM_), and effector memory T cells (T_EM_) using the memory-associated molecule (CD62L) and T cell activation–associated molecule (CD44). Of these, T_N_ (CD44^-^CD62L^+^) is mainly located in the thymic center of the primary or secondary peripheral lymphoid organs in the whole body; T_CM_ (CD44^+^CD62L^+^) is mainly located in the secondary lymphoid organs and is capable of proliferating and differentiating into effector T cells when the body receives antigenic stimulation, with mediated immune protection; and T_EM_ (CD44^+^CD62L^-^) is mostly located in various circulating peripheral tissues and can immediately produce effector factors such as IFN-γ and TNF-α (18, 19). These different T cell subpopulations have their own unique functions and phenotypes, and coordinate with each other in the body to maintain the stability of the adaptive immune environment.

Based on the aforementioned delineation of T cells, Different subgroups of CD8^+^ T cells were detected in the tumor, spleen and draining lymph node tissues of each group in the B16 mouse model. These findings revealed that 80% of CD8^+^ TILs in the tumor tissues were CD44^+^, indicating that most of the CD8^+^ TILs were stimulated by tumor antigens. All three chemotherapeutic agents groups significantly upregulated the proportion of T_CM_ in tumor tissues compared with the PBS control, which was most significant in the DAC group. Chemotherapy promoted the expansion of T_CM_ cells with high proliferative capacity. The proportion of T_EM_ was significantly higher than that of T_CM_ in all treatment groups, indicating that cytotoxic T_EM_ cells dominated the antitumor immune response when the antigen re-invaded (**Supplementary Figures S4A, B**). In the spleen, besides the two previously mentioned populations (T_CM_ and T_EM_), the T_N_ population was present and accounted for a larger percentage. This may be because CD8^+^ T cells were mostly quiescent due to reduced exposure to tumor antigens. The chemotherapeutic agents increased the T_CM_ population, which was consistent with observations in the tumor microenvironment (TME) (**Supplementary Figures S4C, D**). In the tumor-draining lymph nodes, the proportion of T_N_ was the highest in the chemotherapy group (exceeding 80%), while the changes in the proportions of T_CM_ and T_EM_ were reduced compared with the control group (**Supplementary Figures S4E, F**). Previous results indicated that the alterations in the defined CD8^+^ T cell subsets within disparate immune tissues might be associated with their respective immune response statuses. The percentage of T_EM_ increased on sustained antigenic stimulation in the TME. The percentage of T_CM_ significantly increased after chemotherapy in the later stage of the immune response in tumor and spleen tissues, where the inflammatory response had subsided. To a certain extent, chemotherapeutic drugs promote the differentiation of various CD8^+^ T cell subpopulations (T_N_, T_CM_, and T_EM_) in the TME, further contributing to tumor suppression.

### Chemotherapeutic agents significantly expanded CD8^+^ T_TSM_ cells

TdLNs serve as the gateway for tumor metastasis and the sites of adaptive immune responses. They participate in and regulate the systemic antitumor immune response of CD8^+^ T cells (20). Meanwhile, they are closely related to the treatment and prognosis of tumor patients in the clinic (21). However, the differentiation status and effector function of tumor-specific CD8^+^ T cells are affected by the heterogeneity and dynamics of the TME, and the interactions between them are largely unknown. As shown in **Supplementary Figure S4**, chemotherapeutic agents significantly increased the proportion of T_CM_ in the tumor, TdLN, and spleen tissues, mediating sustained immune protection. Recent studies have revealed the presence of a population of antigen-specific memory T cells (CD8^+^ T_TSM_) in the TdLNs with high expression of memory markers (CD62L, CD127, and CD122)and the capacity for self-renewal (5). They can be further polarized into CD62L^+^CD8^+^ Tpex, migrating to the TME to mediate antitumor immune responses.

**Figure 3:**
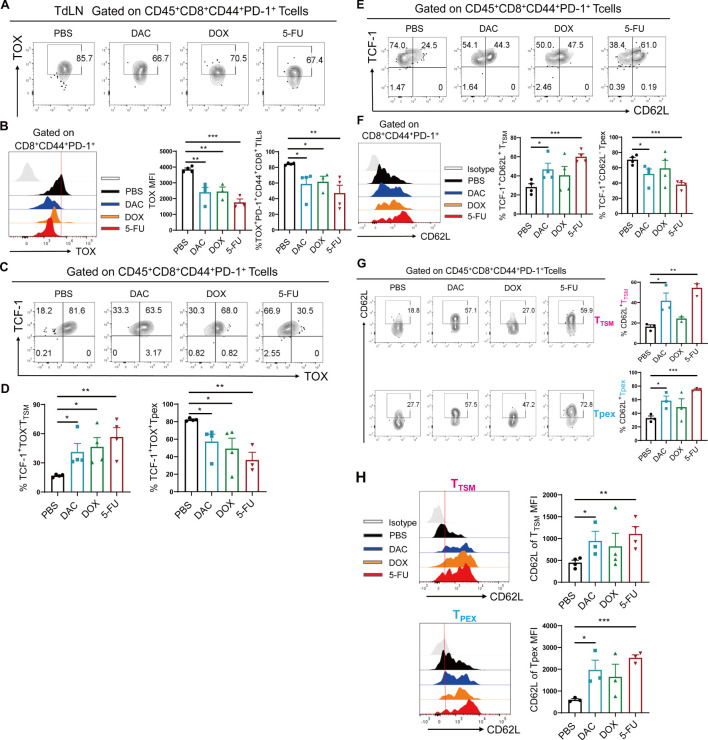
Chemotherapeutic agents significantly expanded CD8^+^ T_TSM_ cells. **(A, B)** Flow cytometry representative plots, MFI value peak plots, and statistical plots of TOX in CD44^+^PD-1^+^CD8^+^ T cells of CT26 tumor-draining lymph nodes. **(C, D)** Flow cytometry representative and statistical plots of TCF-1^+^TOX^-^ T_TSM_ and TCF-1^+^TOX^+^ Tpex cells in TdLNs. **(E, F)** Flow cytometry representative and statistical plots of TCF-1^+^CD62L^+^ T_TSM_ and TCF-1^+^CD62L^-^ Tpex cells in TdLNs and the MFI value peak plot for CD62L in CD44^+^PD-1^+^CD8^+^ T cells. **(G)** Flow cytometry representative and statistical plots of CD62L in T_TSM_ and Tpex cells. **(H)** MFI peak and statistical plots of CD62L in CD8^+^ T_TSM_ and Tpex cells. The data are shown as mean ± SEM. *P < 0.05, **P < 0.01 and ***P < 0.001, using unpaired *t* test, *n* ≥ 3.

Further, we demonstrated that the percentage of TOX and their MFI values were significantly downregulated in the DAC, DOX, and 5-FU treatment groups compared with the PBS control group in the CT26 colorectal cancer model (**Figures 3A, B**), with a higher proportion of TCF-1^+^TOX^-^CD8^+^ T_TSM_ and lower proportion of TCF-1^+^TOX^+^CD8^+^ Tpex cells (**Figures 3C, D**). Further, the proportion of TCF-1^+^CD62L^+^CD8^+^ T_TSM_ cells increased in DAC and 5-FU groups compared with the PBS control group (**Figures 3E, F**). Importantly, the expression of CD62L in TCF-1^+^TOX^-^CD8^+^ T_TSM_ and TCF-1^+^TOX^+^CD8^+^ Tpex cells significantly increased in the chemotherapy groups compared with the PBS control group, with the most significant effect in the DAC and 5-FU treatment groups (**Figures 3G, H**). Overall, these findings indicated that the chemotherapeutic agents inhibited the exhausted phenotype in TdLN CD8^+^ T cells and significantly promoted differentiation, enhancing the memory-like features of stem-like CD8^+^ T_TSM_ cells.

### Chemotherapeutic agents promoted the expansion of CD62L^+^CD8^+^ Tpex

Tumor-specific memory-like CD8^+^ T cells in TdLNs can further differentiate into CD8^+^ Tpex cells, migrating to the TME to exert their corresponding differentiation and effector functions. Both of them are collectively referred to as stem-like CD8+ T cells, which still retain stem-like features in TME (1): self-renewal potential; (2) strong proliferative capacity; and (3) ability to differentiate into Tex^int^ cells. We intended to explore the mechanism underlying the effects of chemotherapeutic agents on the differentiation and expansion of stem-like CD8^+^ T cell subsets in TME. As described previously, TCF-1 and Myb (encoded by *myb*) are the main stem-like markers of CD8^+^ Tpex cells, and CD62L is one of the important markers of memory-associated T cells (22). The expression of the membrane surface molecule Ly108 and the intranuclear molecule TCF-1 is consistent in CD8^+^ Tpex cells (12). Ly108 serves as a substitute marker for TCF-1, whereas CD62L is used to further differentiate Tpex cells into CD62L^+^ Tpex and CD62L^-^ Tpex subpopulations.

**Figure 4:**
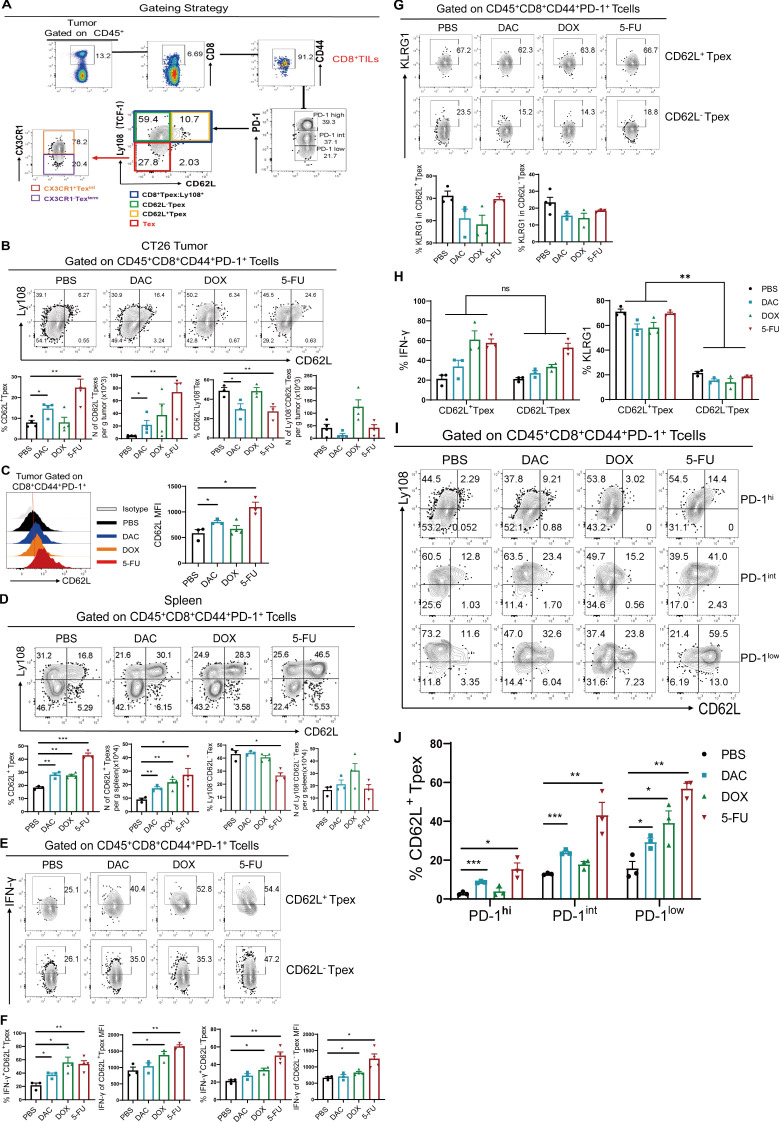
Chemotherapeutic agents promoted the expansion of CD62L^+^CD8^+^ Tpex. **(A)** Flow cytometry gating strategy for identifying CD8^+^ Tex cell subsets in tumor tissues. **(B, C)** Flow and statistical plots of CD62L^+^ Tpex and CD62L^-^ Tpex cells with MFI peak plots of CD62L in CD44^+^PD-1^+^CD8^+^ TIL cells. **(D)** Flow plots and absolute counts of CD62L^+^ Tpex and Ly108^-^CD62L^-^ Tex cells in the spleen. **(E, F)** Flow plots and MFI values of IFN-γ in CD62L^+^ Tpex and CD62L^-^ Tpex cells. **(G)** Flow and statistical plots of exhaustion marker KLRG1 in CD62L^+^ Tpex and CD62L^-^ Tpex cells. **(H)** Summary plot of KLRG1 and INF-γ expression in CD62L^+^ Tpex and CD62L^-^ Tpex cells, respectively, for each treatment group. **(I, J)** Flow and statistical plots of stem-like CD62L^+^ Tpex cells in different PD-1-level populations in CD44^+^PD-1^+^CD8^+^ TILs. The data are shown as mean ± SEM. *P < 0.05, **P < 0.01 and ***P < 0.001, using unpaired *t* test, *n* ≥ 3. ns, means no significance.

First, the protocol described earlier was used in tumor tissues from the CT26-loaded mouse model. The strategy for the four CD8^+^ Tex cell subpopulations is detailed in **Figure 4A**. Both DAC and 5-FU significantly increased the proportion of CD62L^+^CD8^+^ Tpex cells in tumor tissues, the absolute number of cells per gram of tumor, and the MFI value of CD62L cells (**Figures 4B, C**). Similarly, we investigated the distribution of stem-like CD62L^+^ Tpex cells in spleen tissues. We also found that both DAC and 5-FU increased the proportion of CD62L^+^ Tpex cells and their absolute number in spleen tissues compared with the PBS control (**Figure 4D**). In addition, the expression of CD62L and Ly108 in the CD44^+^PD-1^+^CD8^+^ TILs, and the proportion of Ly108^+^ Tpex cells, especially CD62L^+^CD8^+^ Tpex cells, significantly increased in the mouse B16 hormonal tumor model compared with the PBS control group (**Supplementary Figures S5A, B**). These data indicated that DAC and 5-FU both promoted the proliferation and polarization of stem-like CD62L^+^CD8^+^ Tpex cells.

Then, we examined the secretion of the effector molecule IFN-γ in stem-like CD62L^+^ Tpex and CD62L^-^ Tpex cells. The findings demonstrated that only 5-FU significantly promoted the expression of IFN-γ, as indicated by its MFI value, whereas no discernible alteration was evident in the DAC group (**Figures 4E, F**). Recent studies have reported that not only IFN-γ is an effector factor exerting antitumor effects but also its intrinsic type I IFN-γ signaling promotes the differentiation of stem-like CD8^+^ T cells (23, 24). KLRG1, an effector molecule reflecting T cell exhaustion (25), was not differentially expressed in both CD62L^+^ Tpex and CD62L^-^ Tpex cells compared with that in the PBS control group (**Figure 4G**). The aforementioned results suggested that DAC and 5-FU had no effect on the expression of the exhaustion-associated effector marker KLRG1 in stem-like CD62L^+^CD8^+^ Tpex cells, but significantly enhanced the secretion of its effector factor IFN-γ. The expression of KLRG1 was higher than 50% in CD62L^+^ Tpex cells, which was different from that in CD62L^-^Tpex cells (**Figure 4H**). As shown in **Figure 3**, the PD-1 expression in CD44^+^CD8^+^ TIL populations was further analyzed for the percentage of stem-like CD62L^+^ Tpex cells. The results showed that both DAC and 5-FU groups significantly increased the proportion of CD62L^+^ Tpex cells in the PD-1^hi^, PD-1^int^, and PD-1^low^ populations in the CT26 tumor model compared with the PBS control group (**Figures 4I, J**). Only DAC significantly upregulated the proportion of CD62L^+^ Tpex cells in the PD-1^hi^ and PD-1^int^ populations in the B16 melanoma mouse model, and the differences were all statistically significant (**Supplementary Figures S5C, D**). Moreover, the percentage of stem-like CD62L^+^ Tpex was the lowest in the PD-1^hi^ population and higher in the PD-1^int^ or PD-1^low^ population. Overall, DAC and 5-FU significantly promoted the expansion of stem-like CD62L^+^ Tpex cells and their effector functions, and CD62L^+^ Tpex cells were found to have moderate or lower levels of PD-1. DAC and 5-FU promoted the expansion and differentiation of CD62L^+^ Tpex cells by moderately modulating the expression of PD-1, which might be an important basic guideline for the concurrent administration of chemotherapy and anti-PD-1/PD-L1 sequential therapy.

### Chemotherapeutic agents evidently increased CX3CR1^+^ Tex^int^ cells

**Figure 5:**
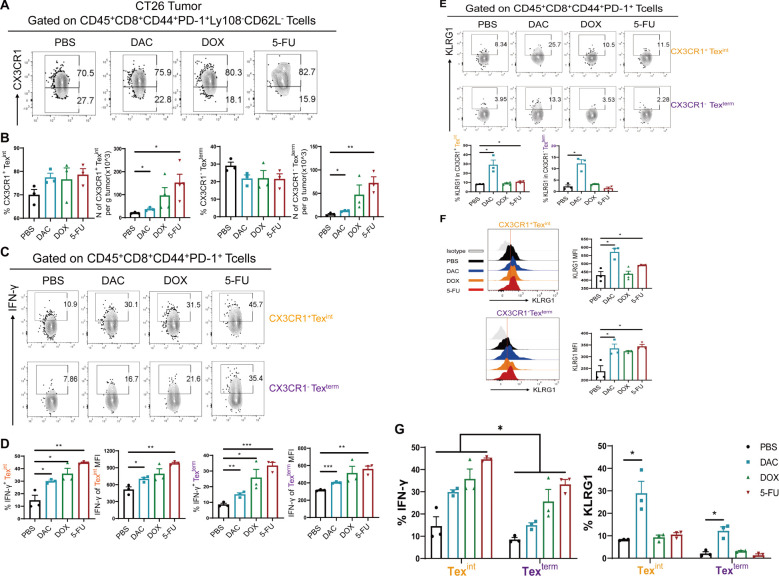
Chemotherapeutic agents evidently increased CX3CR1^+^ Tex^int^ cells. **(A, B)** Flow cytometry analysis and statistical comparison of CX3CR1^+^ Tex^int^ and CX3CR1^-^ Tex^term^ cells in CT26 tumors. **(C, D)** Flow plots of IFN-γ and statistical plots of MFI values in CX3CR1^+^ Tex^int^ and CX3CR1^-^ Tex^term^ cells. **(E, F)** Flow cytometry representative plots and statistical plots of KLRG1 in CX3CR1^+^ Tex^int^ and CX3CR1^-^ Tex^term^ cells. **(G)** Summary plots of the percentages of IFN-γ and KLRG1 in CX3CR1^+^ Tex^int^ and CX3CR1^-^ Tex^term^ cells of each group. The data are shown as mean ± SEM. ^*^ *P* < 0.05, ^**^ *P* < 0.01, and ^***^ *P* < 0.001, using unpaired *t* test, *n* ≥ 3.

CD8^+^ Tex^int^ cells are transitional-state exhaustion effector-like CD8^+^ T cells in the whole CD8^+^ Tex cell subsets, with CX3CR1 as their predominant molecule (26). CD8^+^ Tex^term^ cells differentiate to the terminal phase and rapidly undergo apoptosis. This study aimed to investigate the expression levels of CX3CR1 in Ly108^-^CD62L^-^CD8^+^ Tex cells in the CT26 colorectal cancer model. The results showed that the proportion of CX3CR1^+^ Tex^int^ cells showed an upward trend in the DAC and 5-FU groups. Their absolute numbers were markedly elevated, with the number of CX3CR1^+^ Tex^int^ cells exhibiting an approximately 7.0-fold increase in the 5-FU group compared with a 1.5-fold increase in the DAC group (**Figures 5A, B**), which was consistent with the progression of growth in all groups of colorectal cancers. Moreover, we intended to examine the relative expression of effector factors, specifically IFN-γ and KLRG1, in CX3CR1^+^ Tex^int^ and CX3CR1^-^ Tex^term^ cells. The results showed that the chemotherapy agents significantly enhanced IFN-γ expression in CX3CR1^+^ Tex^int^ and CX3CR1^-^ Tex^term^ cells compared with the control group (**Figures 5C, D**). Concordantly, both DAC and 5-FU groups strongly promoted KLRG1 expression in CX3CR1^+^ Tex^int^ cells, with the most significant effect in the DAC group (**Figures 5E, F**). It was different from the expression of KLRG1 in stem-like CD62L^+^ Tpex and CD62L^-^ Tpex cells. These results, combined with the findings displayed in **Figure 4**, showed that IFN-γ had the highest level of expression in stem-like CD62L^+^ Tpex cells, followed by higher expression in CX3CR1^+^ Tex^int^ cells, and the lowest level of expression in CX3CR1^-^ Tex^term^ cells (**Figure 5G**), which was consistent with the related study reports (12). Meanwhile, the expression of IFN-γ in CX3CR1^+^ Tex^int^ cells exceeded that in CX3CR1^-^ Tex^term^ cells within each group. Only DAC treatment increased the expression of KLRG1 in CX3CR1^+^ Tex^int^ cells and CX3CR1^-^ Tex^term^ cells, which differed from the response observed in stem-like CD8^+^ Tpex cells (**Figure 5G**). These results indicated that stem-like CD62L^+^ Tpex cells and effector-like CX3CR1^+^ Tex^int^ cells were the primary cell populations exerting the immunological response against tumors, and the former could further develop into the latter. We should systematically investigate the regulatory pathway of CD62L^+^ Tpex→Tex^int^→Tex^term^ cell differentiation. The discovery of new strategies for chemotherapeutic agents to promote the expansion and differentiation of CD62L^+^ Tpex cells into antitumor effector-functional Tex^int^ cells may help improve the longevity and efficacy of T cell therapy in clinical cancer.

### Conditional knockout of the transcription factor Eomes in CD8^+^ T cells partially suppressed the promotion of DAC-expanded CD62L^+^ Tpex cells

The transcription factors T-bet/Eomes play a crucial role in regulating the differentiation of T lymphocytes, particularly during CD8^+^ Tex cell differentiation. The differential expression and competitive translocation of T-bet/Eomes within the nucleus is closely related to the functional and terminal differentiation of CD8^+^ T cells, which significantly impacts memory formation and self-renewal ability of CD8^+^ Tex cells (27). Previous research has demonstrated that the transcription factor TCF-1 can sustain a stable population of Tpex cells by upregulating Myb and Eomes expression (22). Therefore, it is hypothesized that chemotherapeutic agents promoting the differentiation of stem-like CD62L^+^ Tpex cells may be closely linked to Eomes.

**Figure 6:**
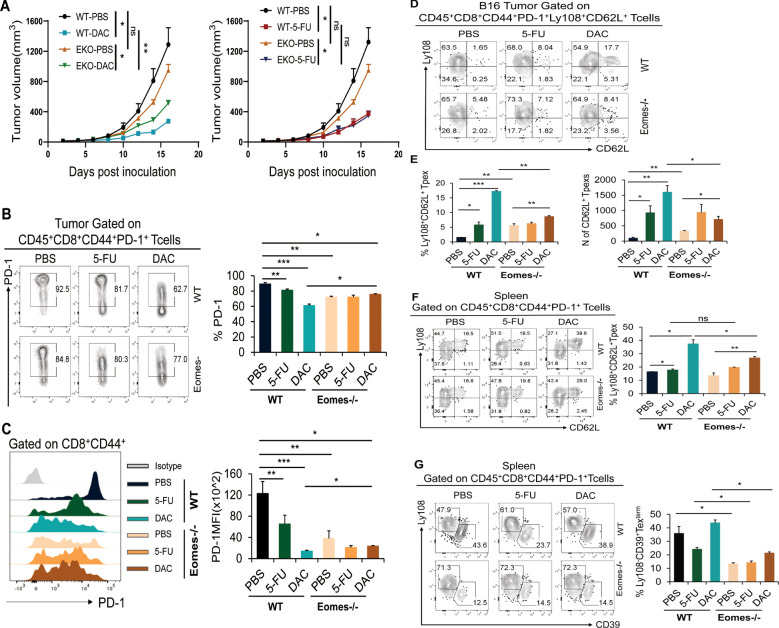
Knockdown of the transcription factor Eomes in CD8^+^ T cells partially suppressed the promotion of DAC-expanded CD62L^+^ Tpex cells. Tumor and spleen tissues of each group were removed to prepare single-cell suspension and analyzed by flow cytometry on day 17 after DAC and 5-FU treatment in WT and Eomes-/- mice. **(A)** Growth curves of the tumors in each group. **(B, C)** Flow representation and statistical diagram of PD-1 and MFI peak diagram of PD-1 in CD44^+^CD8^+^ TIL cells. **(D, E)** Flow cytometry of CD62L^+^ Tpex cell percentage and absolute number of tumors per gram in tumor. **(F)** Flow cytometry representative plots and statistical plots of Ly108^+^CD62L^+^ Tpex cells in the spleen tissue. **(G)** Flow and statistical plots of terminally exhausted Ly108^-^CD39^+^ Tex^term^ cells in the spleen. The data are shown as mean ± SEM. ^*^ *P* < 0.05 and *P < 0.05, **P < 0.01 and ***P < 0.001, using unpaired *t* test, *n* ≥ 3. ns, means no significance.

Therefore, conditional knockout mice (referred to as Eomes-/- mice) were generated by CD4^cre^ × Eomes^fl/fl^ to investigate the impact of Eomes on the expansion and differentiation of stem-like CD8^+^ T cells induced by chemicals. The number and proportion of CD8^+^ Tpex cells in each tissue were assessed using flow cytometry. Consistent with the findings shown in **Figure 2**, both DAC and 5-FU also had inhibitory effects on B16 melanoma growth (**Supplementary Figure S1A**). The conditional knockdown of Eomes in CD8^+^ T cells resulted in accelerated tumor growth in the Eomes-/-_DAC group, significantly reducing inhibition in the DAC group. Conversely, the tumor growth curve in the Eomes-/-_5-FU group closely resembled that in the WT_5-FU group (**Figure 6A**). The aforementioned results indicated that knocking down Eomes modulated the therapeutic effect of DAC but not of 5-FU. Therefore, we further analyzed the expression of PD-1 on CD8^+^ TILs and found that knocking out Eomes abolished the downregulatory effect of DAC on PD-1 expression (**Figures 6B, C**).

This study further analyzed the effect of Eomes knockout in CD8^+^ T cells on the differentiation of stem-like CD62L^+^ Tpex cells. The results demonstrated that the WT_DAC or 5-FU groups exhibited a significant increase in the proportion of stem-like CD62L^+^ Tpex cells and the absolute number of tumors compared with the WT_PBS control group. The proportion of stem-like CD62L^+^ Tpex cells and the absolute number of tumors per gram significantly decreased in the Eomes-/-_DAC group compared with the WT_DAC and WT_5-FU groups but were still higher than those in the Eomes-/- PBS group; however, no such changes were observed in the Eomes-/-_5-FU group (**Figures 6D, E**). We further analyzed the differentiation of CD62L^+^ Tpex cells into stem-like CD8^+^ T cells in spleen tissues. The results showed that the upregulatory effect of CD62L^+^ Tpex cells significantly decreased in the Eomes-/-_DAC group compared with the WT_DAC group. However, the level was still higher than that in the Eomes-/-_PBS group, as seen in tumor tissues (**Figure 6F**). At the same time, the expression of Ly108 and T cell terminal depletion marker CD39 was used to accurately identify CD8^+^ Tex^term^ cell subsets (28). The dates showed that the proportion of CD39^+^ Tex^term^ cells was significantly reduced in Eomes-/-_PBS/DAC/5-FU groups when Eomes in CD8^+^ T cells were specifically knocked out (**Figure 6G**).

Overall, these findings indicated that DAC promoted the differentiation and expansion of stem-like CD62L^+^ Tpex cells in TME and spleen, which was partially dependent on the transcription factor Eomes, and did not modulate the promoting effect of 5-FU. The knockout of transcription factor Eomes significantly affected the differentiation of CD8^+^ Tex^term^ cells.

## Discussion

CD8^+^ TILs in the tumor microenvironment typically exhibit functional exhaustion (29, 30). Exploring effective methods to restore their antitumor function can contribute to tumor eradication. CD8^+^ Tex cells not only have unique characteristics in terms of function, metabolism, transcription, and epigenetics but also form a heterogeneous cell population (31). Recent studies have shown that CD8^+^ Tpex and CD8+ T_TSM_ cells (TdLN-derived stem-like memory cells) both have memory and stem-like characteristics. The two are collectively designated as “stem-like CD8^+^ T cells,” which exhibit robust self-renewal capacity and are capable of proliferating and differentiating into transient effector-like exhausted T cells (Tex^int^) (32). This study mainly explored the roles of the CD62L^+^CD8^+^ Tpex and CD8^+^ T_TSM_ cell subsets in antitumor immunity. CD62L^+^CD8^+^ Tpex cells have unique immunocyte phenotypes and stem cell-like characteristics and play a long-lasting role in tumor immunity. Systematically investigating the regulatory pathways of CD62L^+^ T_TSM_➔CD62L^+^ Tpex➔CD62L^-^Tpex➔CX3CR1^+^ Tex^int^➔CD39^+^ Tex^term^ cell differentiation and discovering new strategies to promote the expansion of CD62L^+^CD8^+^ Tpex cells and their differentiation into Tex^int^ cells with antitumor effect functions can contribute to improving the accuracy and targeting of ICB and ACT treatments.

Clinically, blocking immune checkpoints with antibodies can restore CD8^+^ TIL function and treat tumors more effectively; however, it has not consistently improved survival rates among patients with cancer (33). In addition, conventional chemotherapy is the primary treatment option for cancer patients. Studies show that certain chemotherapeutic agents can enhance the antitumor immune response by increasing the proportion of CD8^+^ T cells. In contrast, standard chemotherapy expands the population of antigen-specific CD8^+^ TILs with minor side effects. Subsequent studies have demonstrated the potential of certain chemical drugs to facilitate the modulation of the antitumor immune response in clinical settings. For instance, the primary administration of 5-FU has been shown to alter the distribution of MDSCs, DCs, and T lymphocytes in the tumor microenvironment, significantly improving the prognosis of patients with advanced gastrointestinal tumors (34). Recent research has validated that the unique niche maintained by cDC1-CD8^+^ T cells activated CD8^+^ Tpex cells systemically, thereby exerting specific antitumor effects (35). It is therefore postulated that 5-FU may exert a modulating influence on the fate of stem-like CD8^+^ Tpex cells, including their differentiation and expansion. Furthermore, among the epigenetically modified chemotherapeutic drugs, the representative of DNA methyltransferase (DNMT1, DNMT3A, and DNMT3B) inhibitors, DAC, has been found to enhance the antitumor effect of CD8^+^ T cells. Studies have confirmed that knocking out DNMT3A in CAR-T cells can prevent cell depletion and enhance the expression levels of key markers including CD28, CCR7, TCF-1, and Lef1 (36, 37). Additionally, the inhibition of *de novo* DNA methylation in CD8^+^ T cells may enhance the efficacy of PD-1 blockade therapy and facilitate the reactivation of antitumor activity in these cells (9). Moreover, low-dose DAC combined with anti-PD-1 treatment can sustain JunD expression within AP-1 family transcription factors to promote the proliferation of CD8+ Tpex cells (38, 39).

Nevertheless, the interaction of 5-FU and DAC with stem-like CD8+ T cells remains unclear. Hence, this study aimed to elucidate the underlying mechanisms of action of 5-FU and DAC. Our findings substantiated that chemotherapeutic drugs could markedly alter the ratio of T_CM_ in the tumor, spleen, and TdLN tissues in the tumor model of B16 melanoma. T_CM_ (CD44^+^CD62L^+^) cell population is mainly located in secondary lymphoid organs and has proliferative capacity. It is capable of rapid proliferation and differentiation into effector T cells, thereby mediating immune protection. Chemotherapeutic drugs can promote the differentiation of various CD8^+^ T cell subsets, eventually forming an antitumor immune microenvironment. Hence, the influence on the differentiation of stem-like CD8^+^ T cell subsets was further analyzed. The results showed that all three chemotherapeutic drugs significantly promoted the differentiation of CD8^+^ T_TSM_ cells and enhanced the expression of CD62L in the draining lymph nodes of mice with colorectal cancer. Meanwhile, the stem-like CD8^+^ T cells in the tumor were analyzed. The results showed that both DAC and 5-FU significantly promoted the expansion of CD62L^+^CD8^+^ Tpex cells and enhanced the expansion and effector function of CX3CR1^+^ Tex^int^ cells in the colorectal cancer tumor model. In conclusion, DAC and 5-FU had the potential to promote the differentiation and expansion of stem-like CD62L^+^CD8^+^ T cells, paving the way for the differentiation of stem-like CD8^+^ T cells. The findings of this study provide a novel theoretical foundation for the chemotherapeutic treatment of colorectal cancer using DAC and 5-FU, accelerating the development of a new regimen of combined chemical drug treatment.

Further, PD-1 expression in stem-like CD62L^+^CD8^+^ Tpex cells is essential. PD-1 levels in CD44^+^CD8^+^ TIL cells are classified into three groups: PD-1^hi^, PD-1^int^, and PD-1^low^. However, stem-like CD62L^+^CD8^+^ Tpex cells are mainly in the PD-1^int^ and PD-1^low^ populations. Both DAC and 5-FU could significantly reduce the PD-1^hi^ population and increase the PD-1^int^ or PD-1^low^ population. Therefore, the present study postulated that DAC and 5-FU might exert a moderate inhibitory effect on TCR and CD28 signals by regulating PD-1, thereby preventing the loss of TCF-1 and Myb expression and avoiding the inhibitory effect of excessive PD-1 expression on the maintenance and self-renewal of CD62L^+^CD8^+^ Tpex cells. This process might help maintain the phenotypic differentiation and stability of CD62L^+^ CD8^+^ Tpex cells.

The T-box family transcription factor member Eomes plays a pivotal role in regulating the differentiation and function of CD8^+^ T cells (40). Eomes shows dynamic changes in the developmental trajectory of the four stages of CD8^+^ Tex cells: Eomes is highly expressed initially in CD8^+^ T cells and again in the terminally exhausted Tex^term^ population. The aforementioned findings prove that the dynamic changes in nuclear Eomes are closely related to the differentiation of exhausted T cells. TCF-1 regulates the relatively high expression of Eomes and plays a role in maintaining naive T cells or exhausted precursor Tpex cells. In contrast, the high expression in terminally exhausted T cells plays a role in protecting T cells from apoptosis. Recent studies have confirmed that the knockout of Eomes can affect the differentiation of CD8^+^ T cells, and Eomes can also affect PD-1 expression. The findings of this Eomes-/- B16 melanoma tumor model were consistent with the aforementioned conjectures. Our findings indicated that DAC and 5-FU had the capacity to markedly enhance the expansion of CD62L^+^ Tpex cells. The knockout of Eomes partially reduced the upregulatory effect of DAC on the proportion and number of stem-like CD62L^+^ Tpex cells. Considering that DAC is an inhibitor of DNMT1, DNMT3A, or DNMT3B transferase, we demonstrated that DAC might partially act on the enhancer or promoter of the Eomes gene to facilitate the differentiation and maintenance of stem-like CD62L^+^ Tpex cells. 5-FU had no effect on Eomes, which might be attributed to its comprehensive effect on CD62L^+^ Tpex cells. It mainly influenced TME-cDC1, indirectly facilitating the differentiation and maintenance of CD62L^+^ Tpex cells; however, its specific mechanism needs further exploration.

In summary, the present study demonstrated that both DAC and 5-FU promoted the differentiation of stem-like CD8^+^ T_TSM_ cells in TdLNs and significantly enhanced the differentiation and expansion of stem-like CD62L^+^CD8^+^ Tpex and CX3CR1^+^ Tex^int^ cells in tumor microenvironment. The knockout of Eomes partially influenced the role of DAC in promoting the differentiation and expansion of stem-like CD8^+^ T cells. This study not only provides new immunological markers for chemotherapy response but also accelerates the development of the clinical combination of chemotherapeutic drugs and T cell immunotherapy, aiming to improve long-term, targeted cancer immunotherapy.

## Materials and methods

### Animals and tumor model

C57BL/6J (B6; H2Kb) and BALB/c mice (6–8 weeks old) were procured from the Shanghai Model Organism Center and housed in the specific pathogen-free facility of the Medical School of Soochow University. All mouse experiments were conducted following the guidelines set forth by the institutional animal care and use committee and with the approval of a protocol. CT26 colorectal cancer cells at a density of 5.0 × 10^5^ cells/50 µL were subcutaneously inoculated into BALB/c mice. On the fifth day after tumor bearing, three chemotherapeutic agents were intraperitoneally injected (i.p.) into the same batch of mice as follows: 5-FU (25 mg/kg, one injection every 2 days for a total of five injections), DOX (1 mg/kg, one injection weekly for a total of two injections), and DAC (0.2 mg/kg, one injection every 2 days for a total of five injections). B16 cell lines were inoculated via intradermal injection (i.d.) into C57BL/6 mice at a dose of 2.0 × 10^5^ cells/50 µL. On the fourth day after subcutaneous tumor bearing, the same batch of mice was treated with the aforementioned three chemotherapeutic agents; also, the tumor size was monitored at 2 to 3-day intervals.

6-8 weeks old CD4^cre^ ×Eomes^flox/flox^ Eomes conditional knock out (EKO) mice, C57BL/6J background, were donated by Prof. Binfeng Lu’s group at the University of Pittsburgh, USA, and housed in the SPF environment of Soochow University Laboratory Animal Center. The chemotherapy treatment of the animal tumor model was as above.

### Mouse tumor tissue digestion and flow cytometry analysis

The tumor-bearing mice were sacrificed after administering chemotherapeutic agents to determine the phenotype and activity of CD8^+^ Tex subsets. Tumor tissues were manually dissociated and minced into small pieces using scissors in 2 mL of cold RPMI 1640 medium without FBS. The tumor masses were then minced and digested with a mixture of 0.33 mg/mL DNase I (Sigma–Aldrich) and 0.25 mg/mL Liberase TL (Roche) in serum-free RPMI at 37.5°C for 30 min. Subsequently, the digested pieces were gently pressed between the frosted edges of two sterile glass slides, and the cell suspension was filtered through a 40-µm cell strainer (BD Biosciences). The immune subsets within the tumor samples were identified using flow cytometry, and the data were subsequently acquired using a FACS flow cytometer (Beckman, CA, USA). Surface staining was conducted with the specified antibodies for 20 min following the manufacturer’s protocols. The intracellular proteins IFN-γ and TNF-α were detected in CD8^+^ TILs following stimulation with Cell Stimulation Cocktail Plus Protein Tran. For intracellular staining of FITC-conjugated anti-IFN-γ(clone number:XMG1.2;catalogue number:505806) and BV785-conjugated anti-TNF-α(clone number:MP6-XT22;catalogue number:506341), the harvested cells were stimulated with PMA (10 ng/mL) and ionomycin (1 µg/mL) for 4 h, followed by a further 1-h incubation with brefeldin A (10 µg/mL). The cells were subjected to intracellular cytokine analysis using antibodies specific for IFN-γ and TNF-α. The antibodies used for FACS included APC-Cy7-conjugated anti-CD45(clone number:30-F11;catalogue number:103116), PerCP/Cy5.5-conjugated anti-CD8(clone number:53-6.7;catalogue number:100734), FITC-conjugated anti-CD44(clone number:IM7;catalogue number:103006), PE-conjugated anti-PD-1(clone number: 29F.1A12), APC-conjugated anti-Ly108(clone number:330-AJ; catalogue number:134160), PC7-conjugated anti-CD62L(clone number:MEL-14;catalogue number:104418), BV421-conjugated anti-CX3CR1(clone number:SA011F11;catalogue number:149023), BV785-conjugated anti-KLRG1(clone number:2F1/KLRG1;catalogue number:138429), Alex Flor 647-conjugated anti-TCF-1(clone number:812145;catalogue number:FAB8224R), and PE-conjugated anti-TOX(clone number:TXRX10;catalogue number:12-6502-82). The data were analyzed using FlowJo V10.0 software (Tree Star Inc., CA, USA).

### Differential expression analysis, GO analysis, and GSEA

The FindMarkers function in Seurat was employed with the Wilcoxon rank-sum test and Bonferroni correction to identify differentially expressed genes (DEGs) in clusters of CD8^+^ TILs. The specific thresholds employed for each set of DEGs and correction methods are detailed in the accompanying legends. GO analysis was performed using clusterProfiler v4.0.5 enriched GO function. The gene set enrichment analysis (GSEA) was conducted using the GSEA function in clusterProfiler v4.0.5, with immunologic signature gene sets sourced from MSigDB (https://www.gsea-msigdb.org/gsea/msigdb/). The mean log4 (fold change) expression values were calculated using the Seurat FindMarkers function.

### Statistical analysis

The data were analyzed with the GraphPad Prism 8.0 software (GraphPad, CA, USA) and presented as mean ± standard error of the mean (SEM). A two-tailed unpaired *t* test was conducted to compare the collected data, with *P <*0.05 indicating a statistically significant difference. Error bars in each figure represent mean ± SEM.

## Data Availability

The raw data supporting the conclusions of this article will be made available by the authors, without undue reservation.

## Supplementary material

Supplementary Figure S1 Chemotherapeutic agents restrained the progression of colorectal cancer and melanoma in mouse and increase the proportion and number of CD8+ TILs and its effector function. The experimental procedure of chemotherapy for B16 melanoma is shown in **Figure 2A**, Chemical therapy was started on the 4th day of subcutaneous tumor loading. **(A)** Physical diagram and tumor growth graph of B16 tumor. **(B, C)** Streaming representation, statistic graph and absolute number of CD45+ immune cells of tumor tissue in B16 melanoma. **(D, E)** Flow-representative graph and statistical graph of absolute number of CD8+ T cells in tumors. Data are shown as mean ± SEM. ***p < 0.001, **p < 0.01, or *p < 0.05 by unpaired t test, n ≥ 3.

Supplementary Figure S2 Chemotherapeutic agents repressed PD-1 expression in CD44+CD8+ TIL cells in tumor tissues. **(A)** Flow plots showing PD-1 expression in CD44+CD8+ TILs of CT26. **(B)** Peak MFI values of PD-1 expression with statistical data. **(C)** Flow plots of distinct PD-1 cell populations with percentage statistics from **(A)**. **(D)** Statistical plot of the flow cytometry results showing PD-1 expression levels in different populations. Summary chart of PD-1 expression percentages in CD44+CD8+ TILs with a heatmap of expression levels. The data are shown as mean ± SEM. **P* < 0.05, ***P* < 0.01, and ****P* < 0.001, using unpaired *t* test, *n* ≥ 3.

Supplementary Figure S3 Chemotherapeutic agents repressed PD-1 expression in CD44+CD8+ TIL cells in tumor tissues. **(A)** Flow-representative graph of PD-1 expression in CD44+CD8+ TILs of B16 melanoma. **(B)** The peak graph of MFI values of PD-1 expression and its percentage statistics. **(C)** Flow representative graphs of distinct PD-1 cell population levels and their percentage statistics in **(A)**. **(D)** The statistical graph of the flow percentage at the level of different PD-1 cell populations. Data are shown as mean ± SEM. ***p < 0.001, **p < 0.01, or *p < 0.05 by unpaired t test, n ≥ 3.

Supplementary Figure S4 Chemotherapeutic agents significantly modified the proportion of TN, TCM, and TEM cells in tumors, draining lymph nodes, and spleen of homozygous mice with melanoma. **(A)** Flow cytometry of TCM and TEM cells in B16 melanoma treated with chemotherapies. **(B)** Total proportion of TCM and TEM cells in tumor and their statistical plot. **(C, D)** Flow cytometry and statistics of TN, TCM, and TEM cells in spleen. **(E, F)** Flow cytometry and statistical data on TN, TCM, and TEM cells in TdLNs. The data are shown as mean ± SEM. **P* < 0.05 and ***P* < 0.01, using unpaired *t* test, *n* ≥ 3.

Supplementary Figure S5 Chemotherapeutic agents promoted the expansion of CD62L+CD8+ Tpex and their characteristics in the tumor microenvironment. **(A, B)** Flow representation and statistical diagram of Ly108+ Tpex, CD62L+ Tpex, CD62L- Tpex and Ly108-CD62L- Tex cells and MFI peak diagram of Ly108 and CD62L in CD44+PD-1+CD8+ TILs cells of B16 melanoma. **(C, D)** Flow representation and statistics of Stem like CD62L+ Tpex and Ly108-CD62L- Tex cells in CD44+PD-1+CD8+ TILs from different PD-1 level populations of B16 melanoma. Data are shown as mean ± SEM. ***p < 0.001, **p < 0.01, or *p < 0.05 by unpaired t test, n ≥ 3.
