# Supplementary material for: Two chemotherapeutic agents expand stem-like CD62L+CD8+ T cells in antitumor immune responses
Source: Front Immunol. 2025 Apr 1;16:1533857. doi: 10.3389/fimmu.2025.1533857 (PMC11996895; doi:10.3389/fimmu.2025.1533857)
Supplement: Supplementary Figure S1 — Chemotherapeutic agents restrained the progression of colorectal cancer and melanoma in mouse and increase the proportion and number of CD8+ TILs and its effector function. The experimental procedure of chemotherapy for B16 melanoma is shown in Figure 2A , Chemical therapy was started on the 4th day of subcutaneous tumor loading. (A) Physical diagram and tumor growth graph of B16 tumor. (B, C) Streaming representation, statistic graph and absolute number of CD45+ immune cells of tumor tissue in B16 melanoma. (D, E) Flow-representative graph and statistical graph of absolute number of CD8+ T cells in tumors. Data are shown as mean ± SEM. ***p < 0.001, **p < 0.01, or *p < 0.05 by unpaired t test, n ≥ 3. [file Presentation1.pptx]

## Slide 1
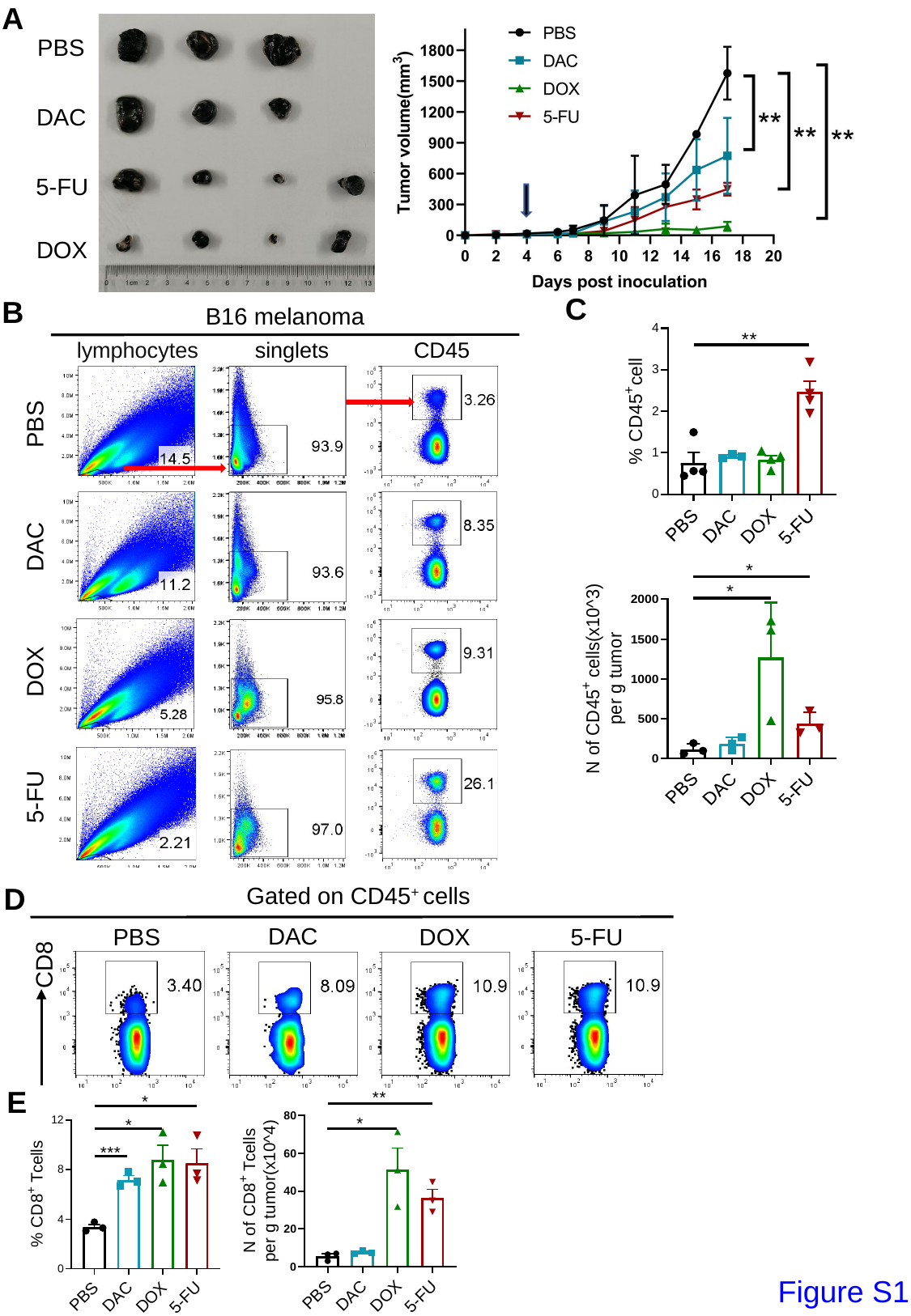

A
PBS
DAC
5-FU
DOX
C
B
B16 melanoma
 lymphocytes
singlets
CD45
PBS
DAC
DOX
5-FU
D
Gated on CD45+ cells
DAC
PBS
5-FU
DOX
CD8
E
Figure S1

## Slide 2
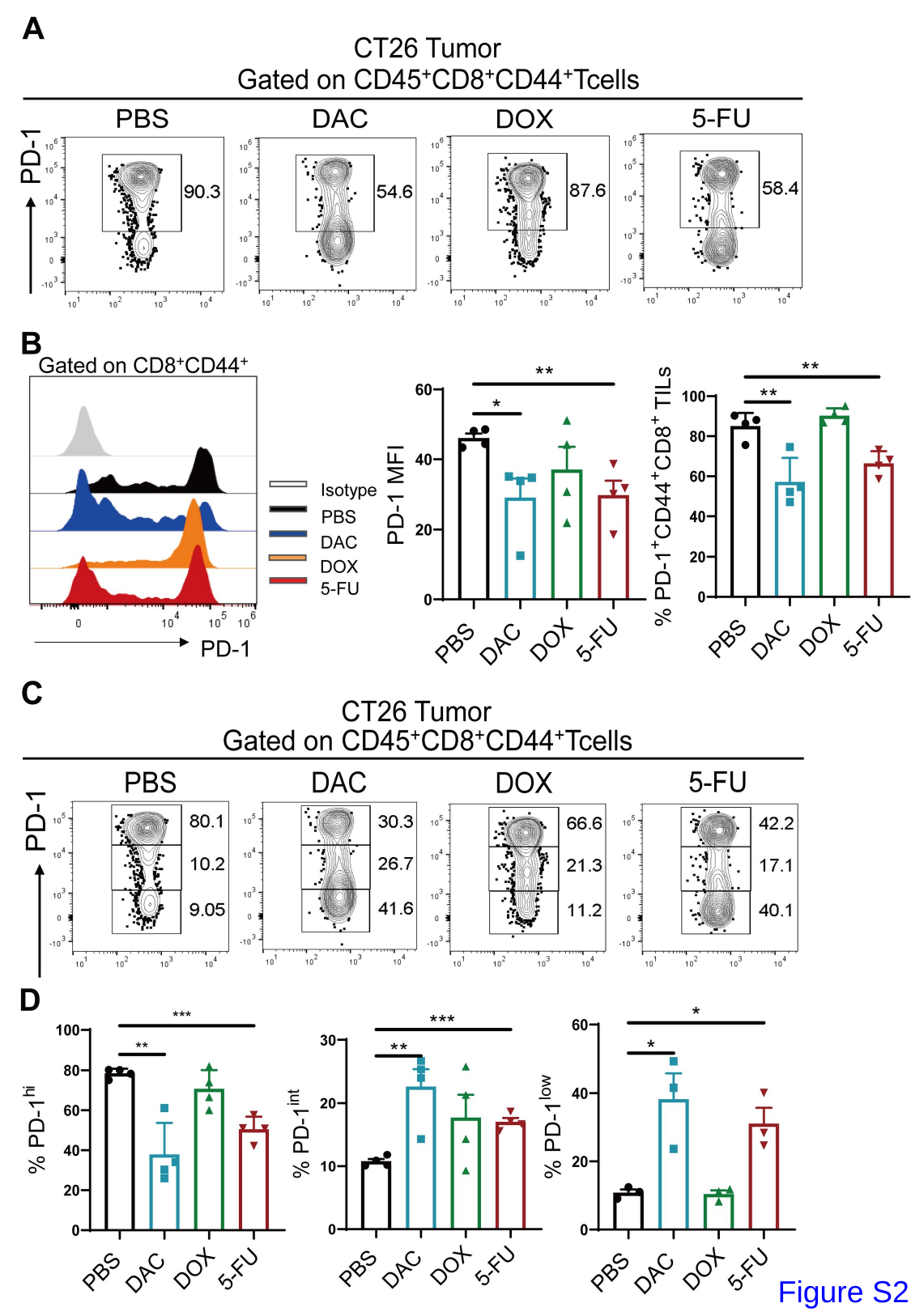

Figure S2

## Slide 3
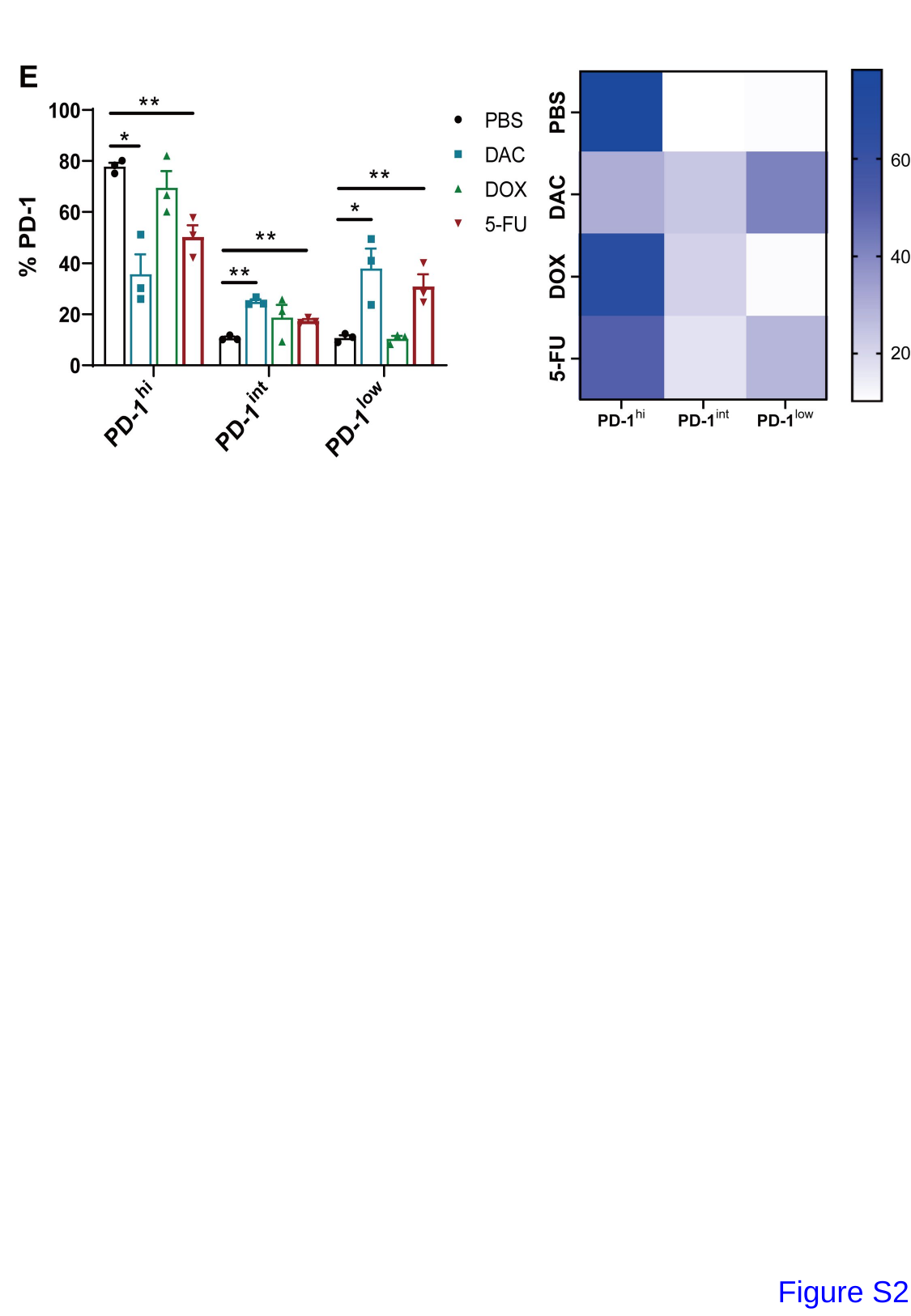

Figure S2

## Slide 4
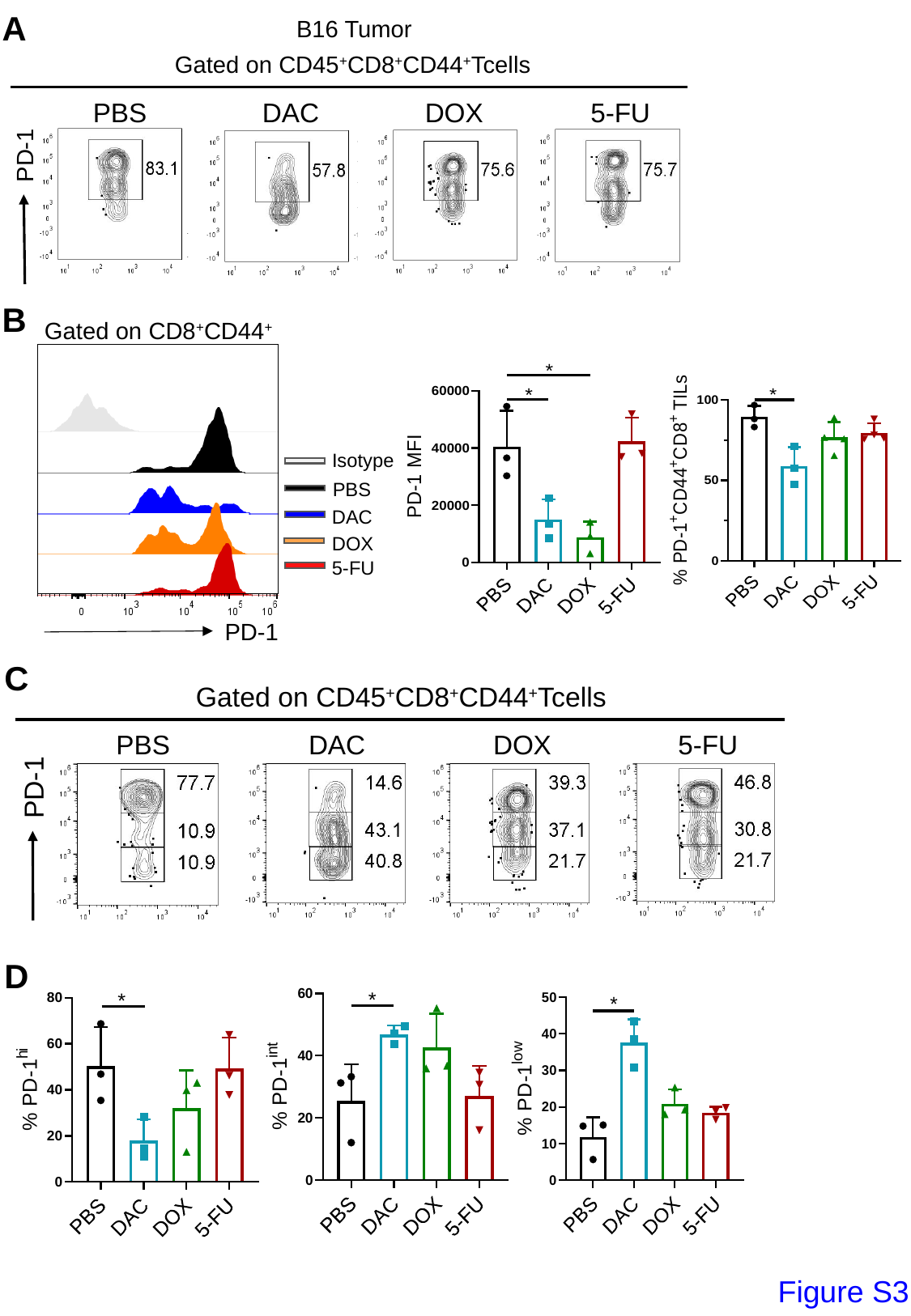

A
B16 Tumor
Gated on CD45+CD8+CD44+Tcells
PBS
DAC
DOX
5-FU
PD-1
B
Gated on CD8+CD44+
Isotype
PBS
DAC
DOX
5-FU
PD-1
C
Gated on CD45+CD8+CD44+Tcells
PBS
DAC
DOX
5-FU
PD-1
D
Figure S3

## Slide 5
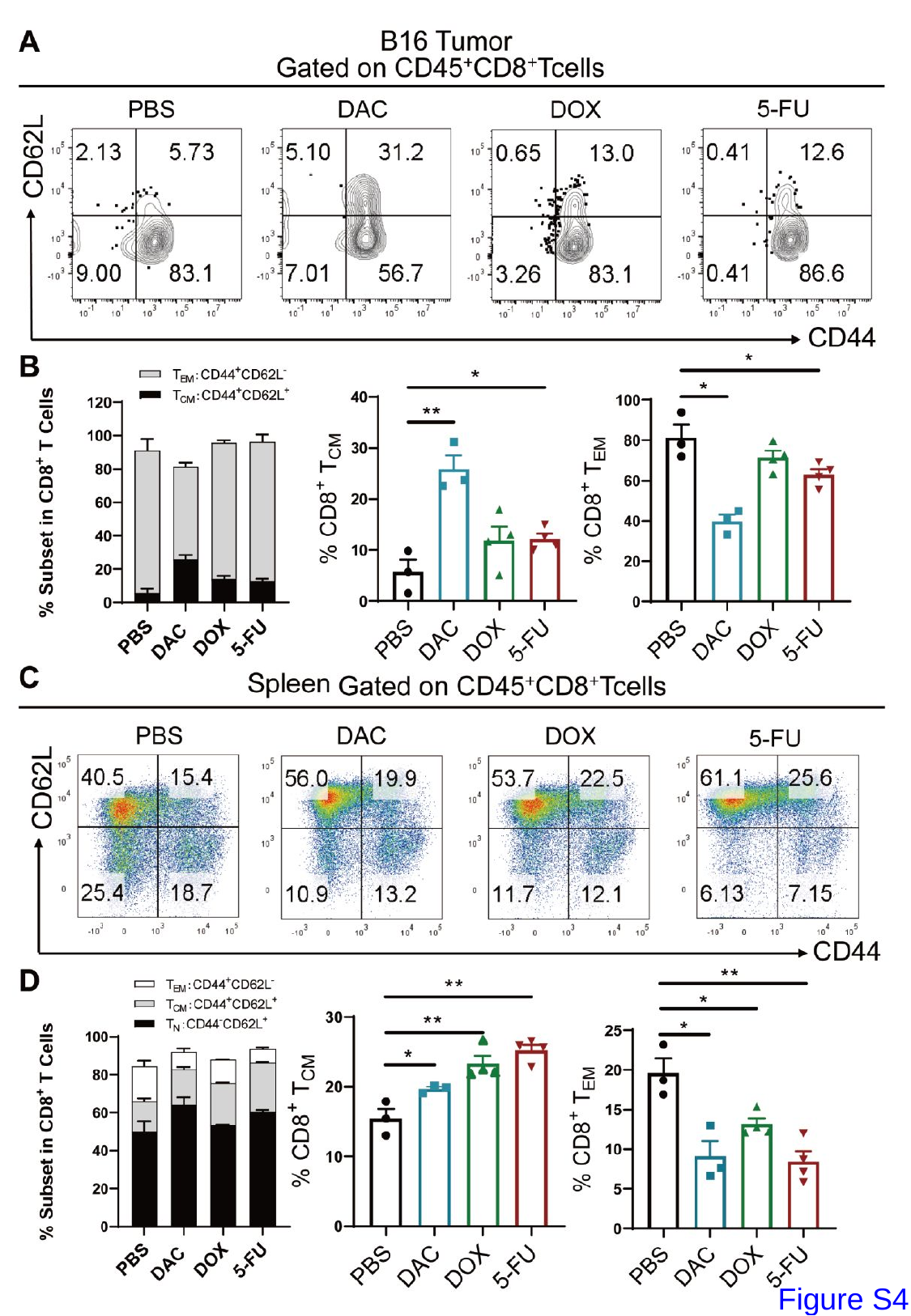

Figure S4

## Slide 6
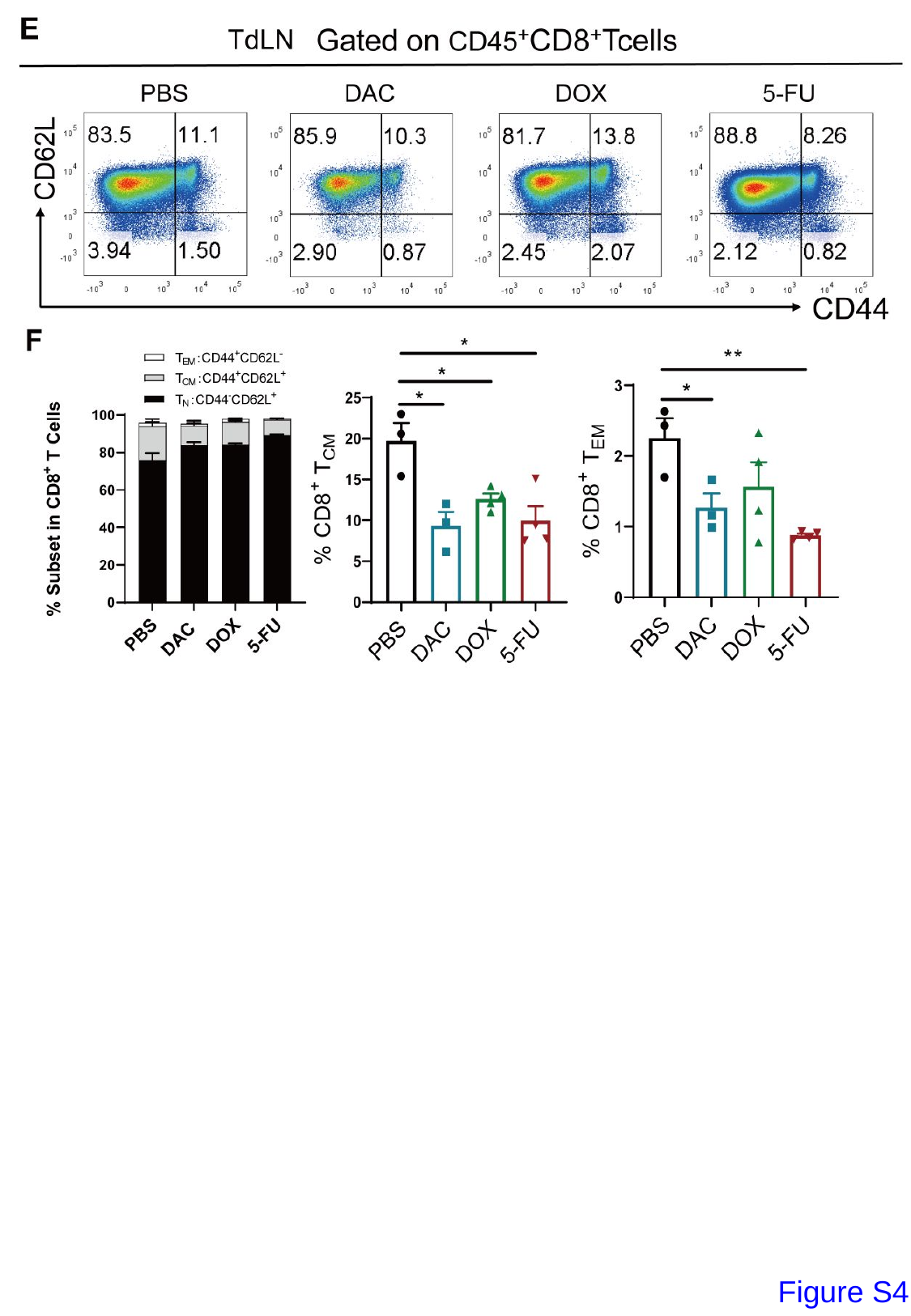

Figure S4

## Slide 7
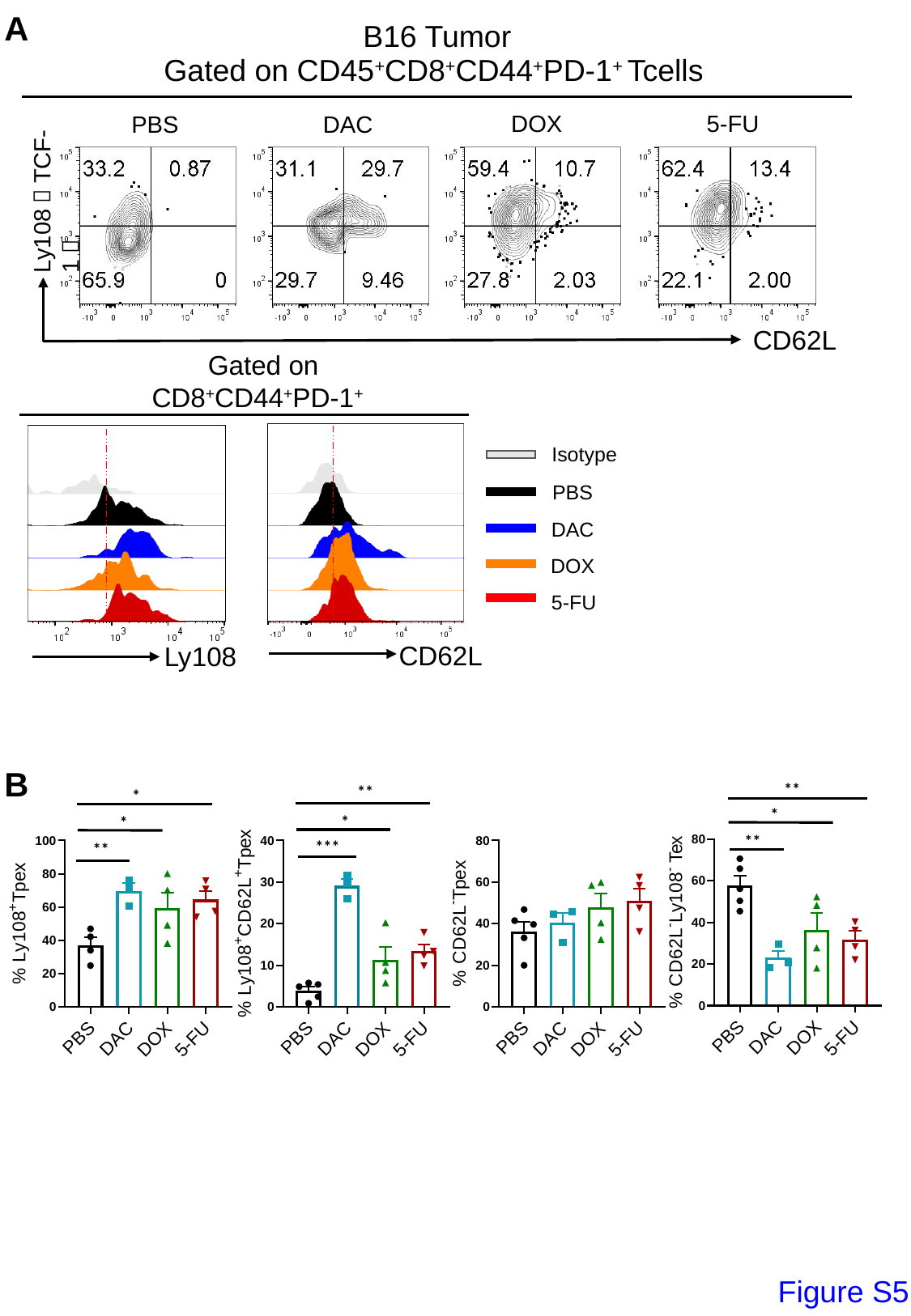

A
B16 Tumor
Gated on CD45+CD8+CD44+PD-1+ Tcells
DOX
5-FU
DAC
PBS
Ly108（TCF-1）
CD62L
 Gated on
 CD8+CD44+PD-1+
Isotype
PBS
DAC
DOX
5-FU
CD62L
Ly108
B
**
**
*
*
*
*
**
***
**
Figure S5

## Slide 8
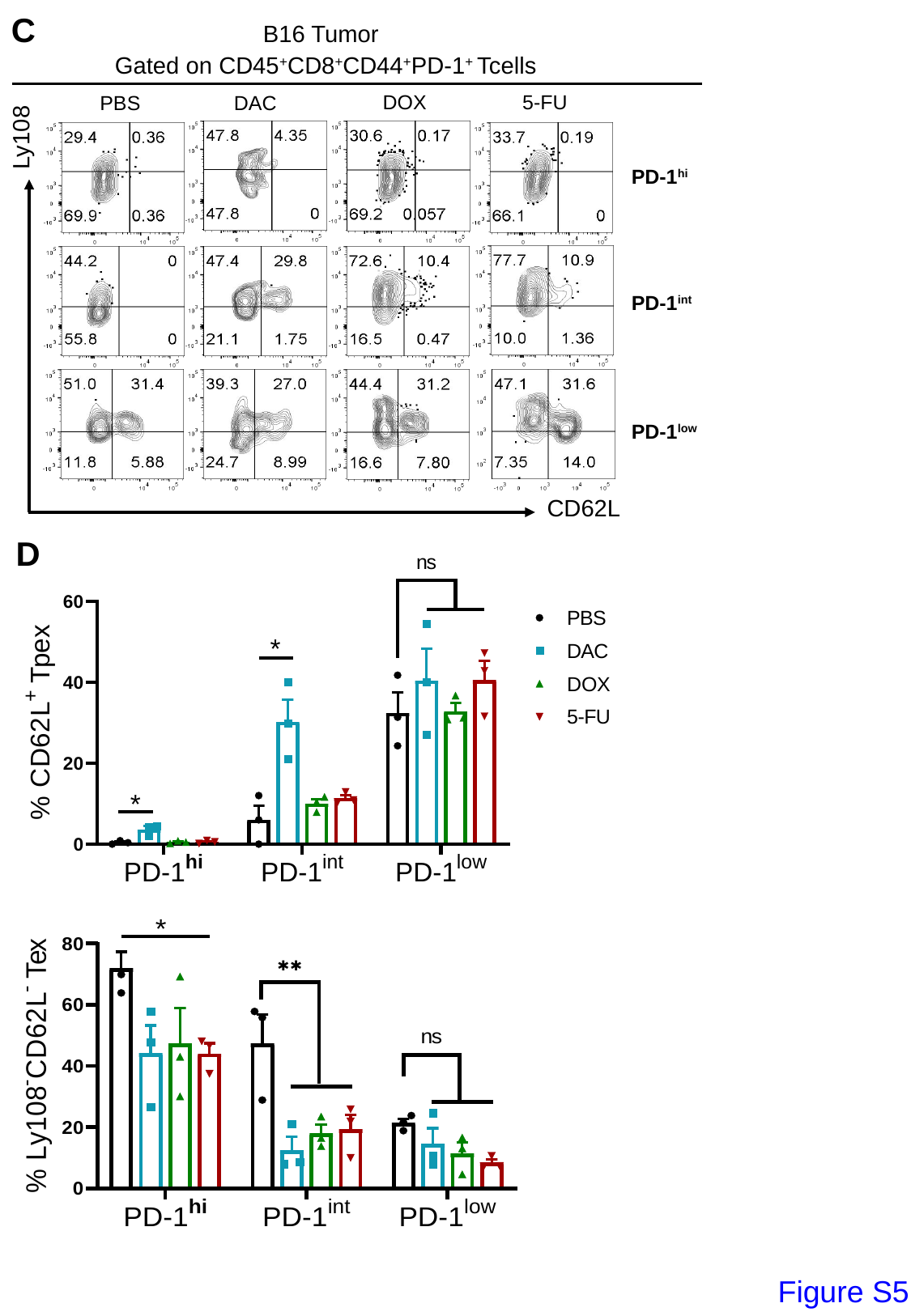

C
B16 Tumor
Gated on CD45+CD8+CD44+PD-1+ Tcells
DOX
5-FU
DAC
PBS
Ly108
PD-1hi
PD-1int
PD-1low
CD62L
D
Figure S5
